# SnS Quantum Dots Enhancing Carbon-Based Hole Transport Layer-Free Visible Photodetectors

**DOI:** 10.3390/nano14110956

**Published:** 2024-05-29

**Authors:** Rui Zhang, Jing Li, Sainan Liao, Shuxin Huang, Chenguang Shen, Mengwei Chen, Yingping Yang

**Affiliations:** Department of Physics, School of Science, Wuhan University of Technology, Wuhan 430070, China; zhangrui123@whut.edu.cn (R.Z.); 272918@whut.edu.cn (J.L.); liaosainan@whut.edu.cn (S.L.); 327320@whut.edu.cn (S.H.); 347475@whut.edu.cn (C.S.)

**Keywords:** SnS quantum dots, perovskite, photodetector, interface modification, heterojunction

## Abstract

The recombination of charges and thermal excitation of carriers at the interface between methylammonium lead iodide perovskite (PVK) and the carbon electrode are crucial factors that affect the optoelectronic performance of carbon-based hole transport layer (HTL)-free perovskite photodetectors. In this work, a method was employed to introduce SnS quantum dots (QDs) on the back surface of perovskite, which passivated the defect states on the back surface of perovskite and addressed the energy-level mismatch issue between perovskite and carbon electrode. Performance testing of the QDs and the photodetector revealed that SnS QDs possess energy-level structures that are well matched with perovskite and have high absorption coefficients. The incorporation of these QDs into the interface layer effectively suppresses the dark current of the photodetector and greatly enhances the utilization of incident light. The experimental results demonstrate that the introduction of SnS QDs reduces the dark current by an order of magnitude compared to the pristine device at 0 V bias and increases the responsivity by 10%. The optimized photodetector exhibits a wide spectral response range (350 nm to 750 nm), high responsivity (0.32 A/W at 500 nm), and high specific detectivity (>1 × 10^12^ Jones).

## 1. Introduction

Perovskite photodetectors have emerged as a revolutionary new photoelectric device and have found applications in various fields, including photonic imaging, optical communications, and optical remote sensing [1,2]. The structure of organic–inorganic metal halide perovskite is typically represented as ABX_3_, where A is the organic or inorganic cation (CH_3_NH_3_^+^, FA^+^ or Cs^+^), B is the divalent metal cation (Pb^2+^ or Sn^2+^), and X is the halogen element (I^−^, Br^−^ or Cl^−^). B-site and X-site atoms form an octahedral structure, while A-site atoms are situated in the interstitial spaces among these octahedra [3,4]. Organic-inorganic metal halide perovskite has advantages including a narrow band gap [5], high absorption coefficient [6], and considerable charge diffusion length [7]. Perovskite photodetectors are commonly classified into three types: photovoltaic, photoconduction, and transistor. Specifically, photovoltaic photodetectors comprise a structure that typically includes a conductive transparent substrate, an electron transport layer (ETL), an absorber layer, a hole transport layer, and metal electrodes [8]. To date, the majority of photodetector researches have concentrated on enhancing the responsivity of photodetectors with complex structures. However, conventional hole transport layer materials (Spiro-OMeTAD) and metal electrodes (gold and silver) not only elevate device fabrication costs but also compromise device stability. This is because of the diffusion of hydrophilic salts within the hole transport layer and the corrosion induced by halogen elements emanating from the metal electrodes [9,10,11,12,13]. Therefore, a carbon-based HTL-free structure has been reported [14,15,16]. By comparing the stability of carbon-based hole transport layer-free photodetectors and photodetectors with spiro-OMeTAD as the hole transport layer, it was found that after being placed in air for 35 days, the performance of carbon-based structure photodetectors only slightly decreased, while the performance of photodetectors with spiro-OMeTAD as the hole transport layer decreased by 50% [14,17]. Carbon-based hole transport layer-free photodetectors exhibit promising application prospects.

However, it has been revealed with further investigation and research that direct contact between the carbon electrode and the perovskite layer induces more interfacial losses. On the back surface of perovskite, many dangling bonds are generated due to the cessation of crystal growth. These dangling bonds form defect recombination centers, causing significant non-radiative recombination of photogenerated carriers at this interface and severe loss of photocurrent [18,19]. Furthermore, the energy-level mismatch between the carbon electrode and perovskite impairs the hole extraction efficiency of the devices [20]. To improve the interface contact between the carbon electrode and the perovskite layer, a strategy involving the integration of additives into the carbon electrode has been suggested. Wang and colleagues showed that the energy-level alignment between the carbon electrode and the perovskite layer was improved by embedding NiO*_x_* nanoparticles in laser-induced graphene thin films [21]. The introduction of NiO*_x_* added new energy levels between the electrode and the perovskite; the conduction band bottom of NiO*_x_* was much higher than that of perovskite, and the valence band top of NiO*_x_* was located between the valence band top of perovskite and the work function of graphene film. This improved the energy alignment. This optimization enhanced the interfacial contact between the two materials. Another alternative optimization strategy is controlling the crystallization of perovskite thin films to achieve a high-performance perovskite absorber layer. Wang et al. [22] incorporated formamide into the perovskite precursor solution to facilitate the formation of prenucleation PbI_2_–DMSO complexes. This method accelerated nucleation and enhanced the crystallinity of the perovskite film, optimizing the energy-level alignment at the interface, thereby enhancing the optoelectronic properties of the device. Incorporating additives at the interface between the carbon electrode and the perovskite layer is considered a potent strategy for improving device performance. This strategy retains the advantages of solution-based photodetector fabrication while circumventing the complexities associated with crystallization and nucleation control.

Quantum dots are a commonly used interface additive. In the past two decades, reports on quantum dot interface passivators have emerged in various fields [23,24]. In recent years, Yang et al. [25] investigated the interfacial challenges in carbon-based HTL-free perovskite photodetectors by implementing a perovskite quantum dot interlayer to bridge the perovskite active layer and the carbon electrode. The incorporation of perovskite QDs into the perovskite film led to an increased contact area between the film and the carbon electrode, enhanced carrier lifetime, diminished defect density, and inhibited non-radiative recombination. This is not the first time that QDs have been added between PVK and carbon electrodes. Prior to this, scholars had already used perovskite QDs as interface passivation [26]. Concurrently, compound semiconductor QDs have undergone extensive study and found wide application in interface and functional layers [27,28,29]. SnS QDs such as IV–VI group compound semiconductor QDs possess advantages such as low cost, non-toxicity, easy accessibility, high absorption coefficient (>10^4^ cm^−1^), and tunable narrow bandgap. Compared to ZnS QDs, SnS QDs have a narrower energy gap and relatively new synthesis methods, providing new possibilities in the field of optoelectronic detection [30,31,32]. This can also be attributed to the propensity of tin to oxidize, which results in the formation of numerous tin vacancies, thereby exhibiting p-type semiconductor characteristics [33]. The multi carriers of p-type materials are holes, which can play a role in transporting holes. At the same time, p-type materials can form a p-p heterojunction with perovskite, which can suppress electron backflow by utilizing the built-in electric field:

(1) $$\mathrm{Sn}\to V(\mathrm{Sn})+2h^{+}+\mathrm{Sn}(s)$$

In this study, SnS QDs were introduced into the interface between the perovskite and the carbon electrode to investigate the influence of varying quantum dot layers on the photodetector performance. The study revealed that the spin-coating of a single quantum dot layer served as an interface passivation agent, effectively reducing the formation of defects between the perovskite and carbon layers. Furthermore, this layer facilitated the rapid and efficient collection and transport of photogenerated holes, which greatly enhanced device performance. Following optimization, the dark current of the perovskite photodetector was reduced by an order of magnitude from 1.16 × 10^−8^ A to 5.64 × 10^−9^ A at 0 V bias, and the responsivity increased by 10% (0.32 A/W at 500 nm), achieving a detectivity (D*) of 10^12^ Jones. These parameters are comparable with previously reported self-powered perovskite photodetectors. The advantage of this device lies in its simple structure and preparation process. We can obtain visible light photodetectors with a detectivity of 1012 Jones at low cost [34,35,36,37,38]. This indicates the significant potential of SnS QDs for application in carbon-based HTL-free perovskite photodetectors.

## 2. Experimental Section

### 2.1. Preparation of Materials and Precursors

1-Octadecene (ODE, >90%), oleic acid (OA, AR), oleylamine (OAm, 80–90%), trioctylphosphine (TOP, 90%), thioacetamide (TAA, 99%), and tin(II) chloride (SnCl_2_, 99%) were purchased from Shanghai Macklin Biochemical Co., Ltd. (Shanghai, China). Acetone (AR), n-hexane (AR), and chlorobenzene (AR) were purchased from China National Pharmaceutical Group Corporation Chemical Reagent Co., Ltd. (Shanghai, China). Ethanol (AR), isopropanol (AR), titanium acetylacetonate (75%), fluorine-doped tin oxide (FTO), and TiO_2_ nanoparticles (30 NR-D) were purchased from Optoelectronics Co., Ltd. (Xi’an, China). Dimethyl sulfoxide (DMSO, 99.7%) and dimethylformamide (DMF, 99.8%) were purchased from Sigma-Aldrich Trading Co., Ltd. (Shanghai, China). Methylammonium iodide (MAI, 99.5%) and lead iodide (PbI_2_, 99.9%) were purchased from Greatcell Solar Materials Co., Ltd. (Queenstown, New South Wales, Australia). Toluene and carbon ink were purchased from Shanghai New Materials Co., Ltd. (Shanghai, China). The model of carbon slurry is CH-8. Grid is 2 × 2 mm^2^, 300 mesh. The mesh material is polyester.

Synthesis of oleic acid-encapsulated stannous sulfide quantum dots was carried out using a conventional hot injection method. The resulting quantum dots were then purified by centrifugation using a mixture of acetone and n-hexane, followed by drying of the precipitate and dispersing it in chlorobenzene at a concentration of 10 mg/mL. PbI_2_ and MAI were dissolved in a mixed solvent of DMSO and DMF with a mass ratio of (13:100), followed by stirring at room temperature for 3 h inside a glove box. The precursor solution for TiO_2_ was prepared by mixing titanium acetylacetonate and ethanol at a volume ratio of 1:19, followed by stirring for 30 min. The TiO_2_ mesoporous layer solution was prepared by mixing TiO_2_ nanoparticles and ethanol in a mass ratio of 1:5, followed by stirring for 12 h.

### 2.2. Device Fabrication

First, the FTO glass was cleaned by ultrasonication in deionized water, acetone, isopropanol, and ethanol for 30 min each. It was then dried using a stream of nitrogen gas and treated with UV ozone irradiation for 20 min to remove surface organic impurities. The precursor solution of TiO_2_ was spin-coated onto the FTO surface using a dynamic spin-coating method at a speed of 4000 rpm for 30 s. It was then dried at 150 °C for 10 min, followed by annealing at 500 °C for 30 min. Next, the mesoporous TiO_2_ nanoparticles were spin-coated onto the dense TiO_2_ layer using a static spin-coating method at a speed of 3500 rpm for 20 s. It was dried at 150 °C for 10 min and annealed at 500 °C for 30 min. The perovskite layer was spin-coated using the anti-solvent method at a speed of 1000 rpm for 10 s, followed by an increase in speed to 4000 rpm for 20 s. When the speed reached 4000 rpm, a rapid injection of toluene solution was performed to crystallize the perovskite and reduce the holes left by the evaporated solvent. It was then dried at 100 °C for 10 min. The interface layer of SnS QDs was obtained by drop-casting 40 μL of SnS quantum dot solution onto the perovskite surface and spinning at 4000 rpm for 30 s. It was dried by annealing at 70 °C for 5 min to remove the chlorobenzene solvent. Finally, the carbon electrode was screen-printed and annealed at 100 °C for 10 min.

### 2.3. Characterization and Device Measurements

The quantum dot morphology and crystalline phase were characterized using transmission electron microscopy (TEM, JEM-2100F, JEOL, Tokyo, Japan) and X-ray diffraction spectroscopy (XRD, D8 Advance, AXS, Billerica, Germany). The absorption of the quantum dots and perovskite thin films was characterized using a UV–vis spectrophotometer (UV-2600, Shimadzu, Tokyo, Japan), while the steady-state photoluminescence (PL, RF-6000, Shimadzu, Tokyo, Japan) spectra of the perovskite thin films were characterized using a fluorescence spectrometer. The section images of the devices were obtained using scanning electron microscopy (SEM, JSM 7500F, JEOL, Tokyo, Japan). The photocurrent and response time were measured using a digital source meter (Keithley, 4200, Solon, OH, USA) and a composite light source (CME-OPS1000, Zhongke Microenergy Co., Ltd., Beijing, China). Electrochemical impedance spectroscopy (EIS, Zahner Company, Kronach, Germany) was performed using an electrochemical workstation under dark conditions and at a bias voltage of 0.8V, with a frequency range of 1 Hz–4 MHz. The quantum efficiency of the devices was characterized using a quantum efficiency testing system (Newport Corporation, Irvine, CA, USA). The surface hydrophobicity was measured using a contact angle goniometer (DSA100, KRÜSS, Hamburg, Germany).

## 3. Results and Discussion

SnS QDs were synthesized and purified via the thermal injection method, as reported in previous literature [39]. Oleic acid (OA), oleylamine (OAm), and trioctylphosphine (TOP) were employed as capping ligands to constrain the growth of the nanoparticles. The synthesis protocol for SnS QDs is detailed in the Section 2.

**Figure 1:**
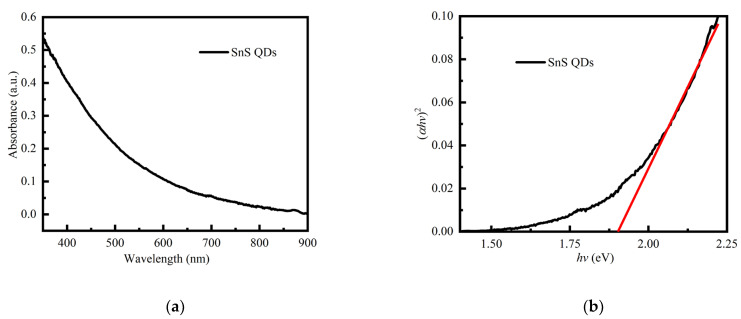

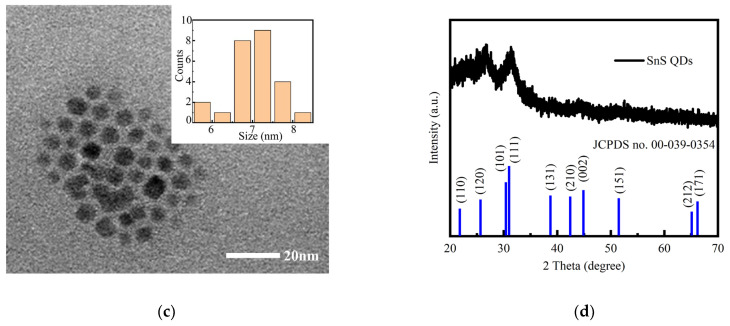
(**a**) UV–vis absorption spectra, (**b**) Tauc plot and fitting line, (**c**) TEM images, and (**d**) XRD patterns of as-synthesized SnS QDs. The inset of (**c**) shows size histogram of SnS QDs.

The optical properties, morphology, and crystal structure of SnS QDs were characterized, with the results presented in Figure 1. Examination of Figure 1a,b reveals that the QDs demonstrate significant absorption within the visible light spectrum. The Tauc plot indicates a direct bandgap value of 1.88 eV. The TEM image shows that the QDs feature a narrow size distribution with an average diameter of 7.15 nm, as shown in Figure 1c. XRD analysis of the sample is illustrated in Figure 1d. Due to the small size of the quantum dots, the peaks are broad and weak, which makes it difficult to analyze their shape and position. However, peaks are present near the (110), (120), (101), and (111) positions of the orthorhombic phase (JCPDS no. 00-039-0354), which is a strong indication that the SnS QDs exhibit this lattice structure. In summary, our study successfully replicated synthesis and yielded high-quality SnS QDs [39,40].

**Figure 2:**
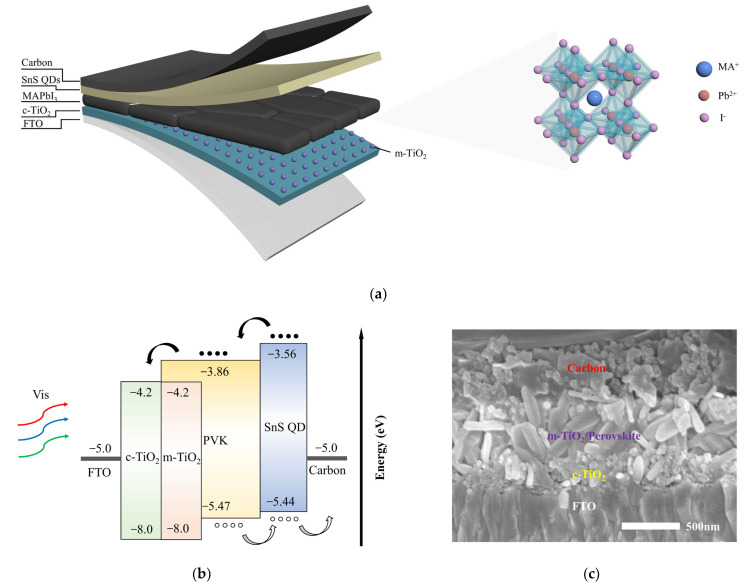
(**a**) Device structure, (**b**) band alignment, and (**c**) cross-sectional SEM of fabricated carbon-based HTM-free photodetector modified with SnS QDs once.

Figure 2 displays the schematic representation, energy band diagram, and cross-sectional SEM image of the photodetector. The device structure comprises fluorine-doped tin oxide (FTO), compact titanium dioxide (c-TiO_2_), mesoporous titanium dioxide (m-TiO_2_), perovskite, SnS QDs, and carbon electrode. The compact TiO_2_ layer has a thickness of approximately 200 nm. Mesoporous TiO_2_ nanoparticles act as nucleation sites for facilitating their growth. In Figure 2c, TiO_2_ nanoparticles are embedded on the front surface of the perovskite film, with a total thickness of approximately 600 nm. Owing to the relatively low concentration of SnS QDs used in the spin-coating process, direct measurement of their thickness presented a challenge. Consequently, we estimated the thickness of the film to be ten nanometers, employing the formula for Newtonian fluids [41,42]. The energy band data were reported in our previous work [43]. The energy band diagram reveals that the addition of SnS QDs solves the band mismatch issue between perovskite and the carbon electrode. The high conduction band of SnS QDs hinders the electron migration towards the SnS QDs layer and reduces the electron–hole recombination at the back surface of the perovskite [44].

**Figure 3:**
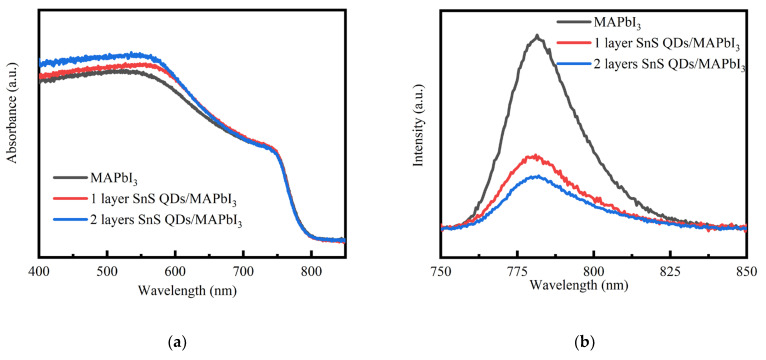

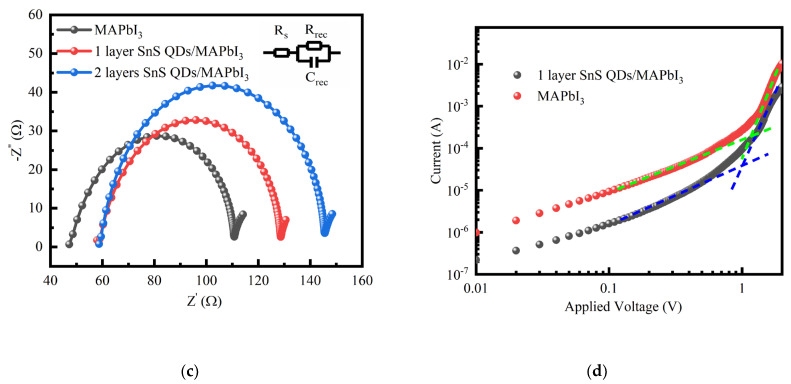
(**a**) UV-vis absorption; (**b**) steady-state PL spectra of the perovskite films modified with SnS QDs for various times; (**c**) EIS data and (**d**) SCLC curves for the pristine and SnS QDs-modified devices and fitting line. The inset of (**c**) shows the simplified equivalent circuit for the impedance spectroscopy analysis.

To investigate the impact of the interface layer of SnS QDs on perovskite thin film performance, a series of tests was conducted, including absorption, photoluminescence, impedance spectroscopy, and space charge limited current (SCLC) measurements. Figure 3a reveals that the quantum dot-modified composite film exhibits enhanced absorption in the visible spectrum, benefitting light collection in photodetectors. This enhancement is attributed to SnS QDs’ strong visible light absorption. SnS QDs absorb and convert the unabsorbed light of perovskite into photocurrent. Using steady-state PL analysis, we further investigated the influence of quantum dots on their ability to extract charges from perovskite layers. Figure 3b demonstrates PL reduction after device optimization. This suggests that photogenerated charge carriers are more efficiently separated and transferred to the SnS QDs layer. And the multi-layered coating yields denser interface, which can enhance the effect of hole extraction [45]. This is why the device optimized with double-layer quantum dots exhibits lower PL values. To investigate defects at the back surface of perovskite, EIS was performed in darkness at a bias voltage of 0.8 V. Figure 3c illustrates the device impedance spectra across a frequency range from 4 Mhz to 1 kHz. Under dark condition, impedance variation at high frequencies reflects the interfacial performance between the carbon electrode and the perovskite. Interface recombination resistance and series resistance were utilized to characterize these performance attributes. Interface recombination resistance mainly indicates the electron–hole recombination rate at the carbon electrode–perovskite interface, with higher interface recombination resistance suggesting enhanced charge carrier extraction efficiency and reduced recombination losses. Upon coating with QDs, there is an increase in the semicircle diameter in the high-frequency domain, corresponding to increased interface recombination resistance. Increased interface recombination resistance suggests that electron–hole recombination at the perovskite/SnS QDs interface becomes less probable, consequently favoring charge extraction.

This results from the diminished recombination centers, attributed to the QDs coordination with the perovskite surface [46]. In addition, we utilized the SCLC method to calculate the defect density, as shown in Figure 3d. The test structure consisted of FTO/PVK/carbon, and the defect density was calculated using the following formula:

(2) $$N_{\mathrm{trap}}=\frac{2\varepsilon_{0}\varepsilon_{r}V_{\mathrm{TFL}}}{qL^{2}}$$

Here, *ε*_0_ is the vacuum permittivity, *ε*_r_ is the relative permittivity, q is the elementary charge, *L* is the thickness of the perovskite film, and *V*_TFL_ is the trap-filled limit voltage. The initial device and the device optimized with SnS QDs have *V*_TFL_ values of 1.18 V and 1.01 V, respectively. After the introduction of SnS QDs, the calculated defect density *N*_trap_ decreases from 1.03 × 10^16^ cm^−3^ to 8.83 × 10^15^ cm^−3^. This suggests that the incorporation of SnS QDs serves to passivate the surface defects in the perovskite materials, thereby reducing non-radiative recombination [47]. From a theoretical perspective, we confirmed through SCLC experiments that the decrease in defect states is not due to a decrease in the probability of radiative recombination. On this basis, the decrease in PL peak could indicate that charge carriers have been extracted into the SnS QDs layer.

**Figure 4:**
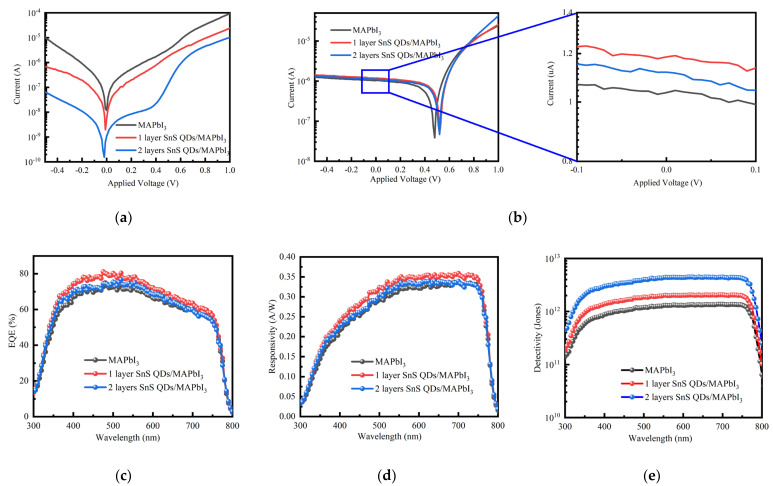
(**a**) Dark I-V curves and (**b**) light I-V curves of different devices. A magnified view of (**b**) is shown for distinguishing the differences in photocurrent of three devices under 0 V bias. (**c**) External quantum efficiency (EQE), (**d**) spectral responsivity, and (**e**) spectral detectivity of the pristine and SnS QDs-modified devices.

Figure 4a,b illustrate the current-voltage (I-V) characteristics of the three devices both in darkness and when exposed to 500 nm wavelength illumination at an intensity of 62 µW/cm^2^. The increasing number of interface quantum dot modifications correlates with a decreasing trend in the dark current at 0 V bias. Specifically, the dark current measured at 0 V bias in the pristine device is 1.16 × 10^−8^ A; after being modified by QDs once and twice, respectively, the dark current values decrease to 5.64 × 10^−9^ A and 1.08 × 10^−9^ A, respectively. The observed reduction in dark current is attributed to the application of quantum dot coating, which serves to passivate the surface dangling bonds of the perovskite. The elimination of surface ligands and the subsequent pairing of dangling bonds on the QD surface suppress thermal excitation in the dark state, thereby reducing the dark current. Additionally, the conduction band minimum of the quantum dot layer is positioned above that of the perovskite, and this difference in energy levels serves to inhibit the reverse migration of electrons [48,49]. The reduction in dark current can be analyzed using the Shockley equation.

(3) $$J_{\mathrm{dark}}(V)=J_{0}[\exp(qV/k_{B}T)-1],$$

*J*_0_ is the reverse saturation current, which is the current of the diode under reverse bias without reaching the breakdown voltage. The electron charge is q = 1.6 × 10^−19^ C, k_B_ is the Boltzmann constant, and *T* is the temperature. The numerical value of *J*_0_ can be expressed by the following equation:

(4) $$J_{0}=\frac{qL_{n}n_{0}}{\tau },$$

*τ* is the minority carrier lifetime, *L*_n_ is the electron diffusion length, and *n*_0_ is the intrinsic electron concentration. When QDs passivate the back surface of perovskite, the reduction in back surface defects could lead to a decrease in the rate of minority carrier surface recombination. This may improve the surface recombination lifetime of minority carriers, thereby increasing the total lifetime of minority carriers and probably suppressing the dark current of the device.

With an increase in the number of quantum dot modifications, the device exhibits an initial increase followed by a subsequent decrease in photocurrent. The photocurrent increases from 1.03 μA to 1.18 μA and 1.12 μA after one and two quantum dot modifications, respectively. This is attributed to the concurrent absorption of visible light by both the perovskite and SnS QDs, followed by their individual transport enabled by the intrinsic electric field, resulting in enhanced harnessing of visible light. SnS QDs exist on the back surface of perovskite materials and do not affect the absorption of incident light by perovskite. SnS QDs have a high absorption coefficient, which is beneficial for them to reabsorb the unabsorbed photons of perovskite. However, the photocurrent decreases when double layers of SnS quantum dots are applied. This reduction may be attributed to an increased amount of organic ligands within the quantum dot layer as well as the induction of defects from partial ligand detachment from the quantum dot surface during the heating process. Additionally, the excessive occurrence of tin vacancies leads to an increase in defect states. Due to the further decrease in device performance after coating with more layers of quantum dots, which is consistent with the above reasons, further layer quantum dot photodetector testing was not conducted. Furthermore, quantum efficiency was performed on the three devices, evaluating their responsivity and the correlation between the spectral detectivity and the wavelength of incident light. As the wavelength increases, the external quantum efficiency (EQE) exhibits a rising trend before subsequently declining in Figure 4c. Specifically, under illumination at 500 nm, the EQE reaches approximately 80%. However, devices incorporating two layers of quantum dots exhibit decreased EQE. This can be attributed to the incorporation of additional quantum dots bound with long-chain organic ligands, which significantly impedes photocurrent transport [50]. The device featuring a single layer of QDs maintains a responsivity of approximately 0.35 A/W across the wavelength range of 550~720 nm, as shown in Figure 4d.

Additionally, throughout the visible spectrum, the device demonstrates a detection ratio of the order of 10^12^ (Figure 4e). In conclusion, a thorough evaluation of the device performance revealed that the single-layer quantum dot photodetector exhibits superior performance.

**Figure 5:**
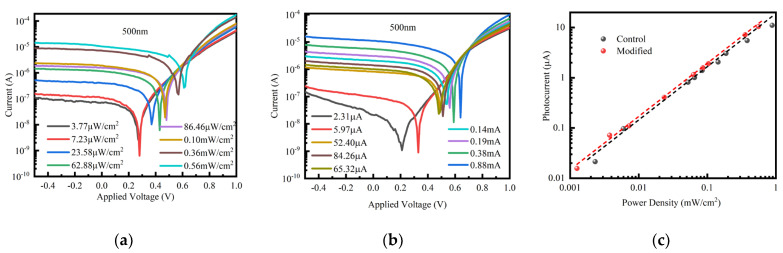
The photocurrent vs. voltage of (**a**) basic devices and (**b**) single-layer QDs optimized devices under 500 nm light irradiation. (**c**) The photocurrent vs. power density of basic devices and single-layer QDs under 500 nm light irradiation.

Significantly, the photocurrent at zero-bias voltage demonstrates a linear dependence on light power density. The photodetector linear response to light power serves as a crucial performance metric, particularly for use in light measurement and imaging applications [51]. From Figure 5, it can be seen that the basic device is no longer able to maintain a linearly increasing photocurrent under light irradiation with a power density of 0.3 mW/cm^2^. The device optimized by single-layer QDs is still in a linear growth state within this range. This may be because when the light intensity increases to a certain extent, the defects on the back surface of perovskite enhance the capture effect of photogenerated carriers, making it unable to maintain a linear growth trend. After quantum dot modification, defects on the back surface of perovskite are passivated, and photogenerated carriers can be effectively transported. After device optimization, the LDR exhibits an increase. Through calculation, the LDR of the device increased from 40 dB to 60 dB after QD optimization.

**Figure 6:**
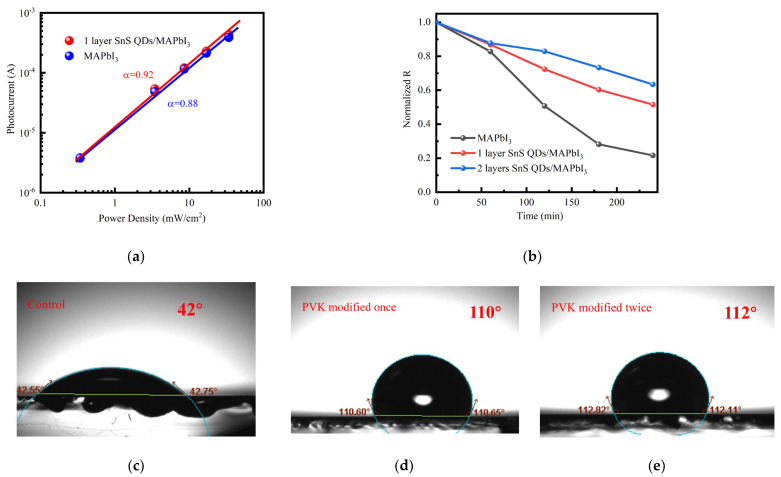
(**a**) Light intensity-dependent photocurrent curves of the pristine and device modified with SnS QDs once. (**b**) Stability testing of three devices. Water contact angle images of the (**c**) perovskite film and the SnS QDs films deposited on the perovskite substrate (**d**) once and (**e**) twice.

Similar to conventional photodetectors, the device still exhibits a saturation state when the light power is too high, and the photocurrent cannot continue to maintain a linear growth trend. To investigate the charge transfer dynamics within the detector under saturation conditions, we performed *J*_sc_ measurements as a function of light intensity. The correlation between *J*_sc_ and light intensity is described by the subsequent equation [52]:

(5) $$J_{sc}\propto I^{\alpha }(\alpha \leq 1)$$

where *α* is an empirical parameter. An *α* value approaching 1 indicates minimal bimolecular recombination under short-circuit conditions. As depicted in Figure 6a, post optimization, the *α* value of the device increased from 0.88 to 0.92. This implies that, before optimization, the device exhibited considerable photogenerated charge carrier activity, trap state presence, and carrier recombination. The optimized device has improved its performance in these parts.

Compared to devices with the same structure, the photodetector in this work has higher responsivity and detectivity in Table 1. In addition, device stability is a critical factor influencing applications. Perovskite materials will quickly decompose into lead iodide when in contact with water, which will cause them to lose their photoelectric response characteristics. Conducting hydrophobic angle testing on it is an indirect method to test its water stability. As demonstrated in Figure 6c–e, by applying a coating of QDs, we successfully enhanced the device surface hydrophobicity. Following the application of single and double layers of QDs, the contact angles on the device’s surface increased to 110° and 113° from 43°, respectively. The increase in hydrophobic angle indicates that the surface of the device has a repulsive effect on water, making it difficult for water vapor to enter the interior of the device. This demonstrates that QDs modification significantly enhances the device hydrophobicity, inhibiting water decomposition reactions and improving the device wet stability. To verify its results, we conducted stability tests on it in 33 °C, 93% RH environments. From Figure 6b, it can be seen that with the increase in time, the performance of the basic device decreases the fastest. After 240 min, the optimized device still maintained 50% of its initial performance, while the performance of the basic device decreased to 20% of its initial performance. This can be attributed to the intrinsically high hydrophobicity of the SnS material, effectively decelerating water vapor diffusion, protecting the perovskite layer, and augmenting the device stability [53,54]. The hydrophobicity of SnS quantum dots may be due to capillary effects counter-balance bulk properties. Compared to optoelectronic devices optimized through P3HT, the performance degradation is almost the same (a 10% decrease in current exposed to 90% RH) [55].

## 4. Conclusions

In this work, we successfully fabricated SnS QDs/MAPbI_3_ heterojunction photodetectors. SnS QDs were deposited on the back surface of the perovskite film by employing the spin-coating technique, wherein these QDs served multiple functions: the modification of interfaces, the enhancement of light absorption, and the facilitation of hole extraction. Owing to these factors, the SnS QDs/MAPbI_3_ heterojunction photodetectors have high responsivity (0.32 A/W at 500 nm) and a superior detectivity (exceeding 1 × 10^12^ Jones) in a self-powered mode. Compared with the pristine device, responsivity improved by 10%, and the detectivity achieved a superior standard. The findings suggest that the use of SnS QDs as an interfacial layer constitutes a promising approach to enhancing the performance of carbon-based HTL-free photodetectors. In summary, the integration of SnS QDs as an interfacial layer presents considerable potential for application within carbon-based HTL-free photodetector technology, offering an efficient means to enhance their performance.

## Figures and Tables

**Table 1:** Summary of performance parameters and device structure of photodetectors.

Device Structure	R(A/W) 500 nm	D* (Jones)	τ_rise_/τ_fall_	Ref.
FTO/PEDOT: PSS + Ag NPs/MAPbI_3_/Al	0.25	1.5 × 10^11^	110 ms/72 ms	[56]
ITO/NiO*_x_*/Pb-Sn Perovskite/C_60_/TmPyPB/Ag	0.32	>1 × 10^12^	14 μs/14 μs	[57]
ITO/NiO*_x_*/PbS QDs/MAPbI_3_/PC_61_BM/Ag	0.34	5.6 × 10^11^	3.5 μs/35 μs	[58]
FTO/TiO_2_/MAPbI_3_ PNCs/PTAA/MoO_3_/Ag	0.27	2 × 10^11^	46 ms/47 ms	[59]
FTO/TiO_2_/m-TiO_2_/MAPbI_3_/SnS QDs/Carbon	0.32	1.9 × 10^12^	47 ms/43 ms	This work

## Data Availability

Data are contained within the article.
